# Importance of Coagulation Factors as Critical Components of Premature Cardiovascular Disease in Familial Hypercholesterolemia

**DOI:** 10.3390/ijms23169146

**Published:** 2022-08-15

**Authors:** Uffe Ravnskov, Michel de Lorgeril, Malcolm Kendrick, David M. Diamond

**Affiliations:** 1 Independent Researcher, Magle Stora Kyrkogata 9, 22350 Lund, Sweden; 2 Laboratoire Coeur et Nutrition, TIMC-CNRS, Faculté de Médecine et Pharmacie-Université Grenoble Alpes, 38000 Grenoble, France; 3 East Cheshire Trust, Macclesfield District General Hospital, Macclesfield SK10 3BL, UK; 4 Departments of Psychology, University of South Florida, Tampa, FL 33620, USA

**Keywords:** familial hypercholesterolemia, low-density-lipoprotein cholesterol, coagulation, cardiovascular, lipoprotein (a) (Lp(a)), probucol, coronary artery calcium, fibrinogen, factor VIII, thrombocytes, diabetes

## Abstract

For almost a century, familial hypercholesterolemia (FH) has been considered a serious disease, causing atherosclerosis, cardiovascular disease, and ischemic stroke. Closely related to this is the widespread acceptance that its cause is greatly increased low-density-lipoprotein cholesterol (LDL-C). However, numerous observations and experiments in this field are in conflict with Bradford Hill’s criteria for causality. For instance, those with FH demonstrate no association between LDL-C and the degree of atherosclerosis; coronary artery calcium (CAC) shows no or an inverse association with LDL-C, and on average, the life span of those with FH is about the same as the surrounding population. Furthermore, no controlled, randomized cholesterol-lowering trial restricted to those with FH has demonstrated a positive outcome. On the other hand, a number of studies suggest that increased thrombogenic factors—either procoagulant or those that lead to high platelet reactivity—may be the primary risk factors in FH. Those individuals who die prematurely have either higher lipoprotein (a) (Lp(a)), higher factor VIII and/or higher fibrinogen compared with those with a normal lifespan, whereas their LDL-C does not differ. **Conclusions:** Many observational and experimental studies have demonstrated that high LDL-C cannot be the cause of premature cardiovascular mortality among people with FH. The number who die early is also much smaller than expected. Apparently, some individuals with FH may have inherited other, more important risk factors than a high LDL-C. In accordance with this, our review has shown that increased coagulation factors are the commonest cause, but there may be other ones as well.

## 1. Introduction

Premature CVD among people with FH is considered to be caused by their high LDL-C. In 1983, Goldstein and Brown were honored with the Nobel Prize for their research on FH, which appeared to confirm the role of raised LDL-C in CVD. However, what they established was not that high LDL-C was the cause of early CVD, but simply that individuals with FH have an inherited defect in the gene encoding the cells’ LDL receptors, which remove LDL from the blood [1].

Many studies have demonstrated that cardiovascular disease (CVD) is associated with high total or LDL-cholesterol, but association is not the same as causation; many other studies have shown that the cholesterol hypothesis is unable to satisfy any of the Bradford Hill criteria for causality [2]. For example, people with low cholesterol have the same degree of atherosclerosis as people with high cholesterol and there is no exposure-response in cholesterol-lowering trials [2]. In addition, more than 30 studies have demonstrated that elderly people with high LDL-C have an equal or greater lifespan than elderly people with normal or low LDL-C and no study has shown the opposite [3,4]. Several studies have also shown that high total cholesterol is equally benign in the elderly population [5]. The positive association between cholesterol and CVD in young and middle-aged people may instead be driven by mental stress because it has been demonstrated that mental stress can raise cholesterol by a substantial amount [6,7] and may, in turn, cause CVD in another way [8]. A further support of our view is that none of the anti-CETP and anti-PCSK9 trials have succeeded with lowering total or CVD mortality, although these drugs are able to lower LDL-C much more than the statins [9].

In a previous review [10], we have pointed out many contradictions to the general consensus about FH. Here, we present a more detailed analysis of the many studies which have shown that only a minority of people with FH die prematurely, and that those who die early have inherited higher levels of various coagulation factors, whereas their LDL-C do not differ from those who have a normal length of life.

## 2. The Contradictions

**Atherosclerosis is not associated with LDL-C:** If a high LDL-C is the primary cause of atherosclerosis, those with the highest values should become more atherosclerotic than those with low values. However, in a previous review [2], we noted four studies in the non-FH population which demonstrate that those with low LDL-C have the same amount of atherosclerosis as those with high values. Furthermore, a study using coronary artery calcium (CAC) scanning of 23 413 middle-aged patients with CVD showed that the degree of CAC was inversely associated with LDL-C [11]. In accordance with this, five studies on individuals with FH found that LDL-C is not associated with degree of atherosclerosis or with intima-media thickness [12,13,14,15,16].

**On average, people with FH live just as long or longer as other people:** It is widely believed that people with FH have a significantly reduced lifespan. However, the clinical observations on FH have been almost exclusively based on studies of people with FH who were selected because of their family history of CVD or who already suffer from heart disease. This has introduced a significant bias. The reality is that two unbiased studies have shown that on average, people with FH have lifespans comparable to the surrounding population. For instance, in a study by the Simon Broome Register group, 526 FH-individuals aged 20–74 years were followed for about four years [17]. Mortality during these four years was higher among those below age 60, but lower among the elderly. In a Norwegian study, 4688 FH-individuals aged 0–80 years were followed from 1992 to 2010 [18]. During that time, cardiovascular mortality was higher than in the general population, but total mortality was lower, although not with statistical significance. Furthermore, before the year 1900, the life span of people with 50% risk of having FH was just as long or longer than in the general population [19]. At that time, infectious diseases were the commonest causes of death. The reason why the lives of people with FH on average are just as long or longer than those of other people may be that LDL participates in the immune system by adhering to and inactivating almost all types of microorganisms and their toxic products, a little-known fact although it has been documented in various ways by more than a dozen research groups [20].

Oxidized LDL-C is considered as a cause of atherosclerosis because it is elevated in patients with CVD, but there is another, more likely explanation. As the macrophages inactivate microorganisms by oxidation, and as all microorganisms are covered with LDL molecules, these molecules may be oxidized as well. The finding that oxidized LDL-C is higher among people with FH and CVD than among their unaffected relatives and among normolipaemic subjects [21] may be a sign of an ongoing infection, not one of CVD.

**No LDL-C difference exists between FH individuals with and without CVD:** According to the cholesterol guidelines, LDL-C should be lowered as much as possible, particularly in FH individuals. However, no cholesterol-lowering trial has demonstrated exposure-response [2]. In a review of 16 angiographic cholesterol-lowering trials where the authors had calculated exposure–response, a correlation was only present in one of them, and in that trial, the only cholesterol lowering treatment was exercise [5]. A review of more than 30 statin-trials found that exposure-response was present [22]. However, in their analysis, the authors only used data from twelve of the trials. If data from all trials are combined, the exposure-response is no longer present [2].

As the level of LDL-C in FH varies considerably, those who suffer from CVD should have higher LDL-C and die earlier than those with the lowest values. A number of studies have shown that LDL-C and the age of those with and without CVD and without lipid lowering treatment did not differ significantly. In most of these studies, many of the participants had been on statin treatment for several years, which may have biased the results. However, in five studies including seven cohorts of FH individuals without cholesterol-lowering treatment, the mean LDL-C was only higher among those with CVD in one of the cohorts (Table 1) [23,24,25,26,27].

**No cholesterol-lowering trial has lowered the risk of CVD of people with FH:** In our previous review of FH [10], we identified ten randomized, controlled cholesterol-lowering trials that included individuals with FH only. None of them succeeded with lowering CHD mortality, total mortality, non-fatal CHD or CVD events. However, nine of the trials were ended after only 1–2 years of treatment, but one of them, where patients with ileal bypass were compared with usual treatment, continued for ten years. Although LDL-C was much lower in the ileal bypass group (360 vs. 468 mg/dL), the number of fatal and non-fatal events was almost identical in the two groups [28]. According to PubMed, many cholesterol-lowering trials of FH individuals have been performed since the publication of our review, but none of them have reported clinical outcomes.

**Other possible causes:** Why, though, do some FH individuals develop premature CVD? The most likely answer is that they have inherited other, more important risk factors, expressed by genes associated with FH genotypes. If this is the case, some of those with normal lipid levels in families with FH should also have a greater risk of premature CVD compared to the surrounding population. Indeed, this has been shown in a study of FH kindred [29]. The lifespan of 40 members was analyzed and it showed that men with FH lived to virtually the same age as relatives without FH, and women with FH lived six years longer than those without FH.

In reality, it has been demonstrated that among FH individuals, several factors are more closely associated with the risk of CVD than LDL-C, and they may indeed be causal. The commonest and most completely documented are inborn or acquired errors of the coagulation system and/or other thrombogenic factors, such as increased platelet reactivity.

**Coagulation factors and platelet reactivity:** In a study of platelet function in 17 FH subjects and 26 non-FH subjects, the aggregation ability of the platelets from those with FH was significantly increased [30]. The same effect was noted in a study where the authors also noted that if they incubated washed platelets from non-FH subjects with plasma from FH subjects, the platelets became almost as sensitive [31]. On the other hand, washed platelets from FH subjects had a significant decrease in activity. These findings strongly suggest that the increased platelet activation seen in FH patients may be induced by other plasma constituents.

Furthermore, plasma fibrinogen and factor VIII levels are significantly higher among those FH individuals who develop CVD, with no measurable difference in the levels of LDL-C or any other lipid [32]. It has also been found that in response to adenosine 5-diphosphate, collagen or thrombin, platelets from FH subjects have a significantly increased binding affinity to 125^l^-fibrinogen compared with platelets from non-FH individuals [33].

In a study where the authors compared 164 FH subjects with and without CVD and without lipid-lowering therapy with 160 normolipidemic controls, the mean platelet volume was significantly higher in the FH subjects [34], and it has been shown that larger platelets are more reactive and thereby more prone to adhesion and aggregation [35].

Other factors may also increase coagulability. In an analysis of DNA extracted from peripheral blood leukocytes in nearly two thousand FH individuals, polymorphism in the prothrombin gene was strongly associated with CVD risk [27], and polymorphism of the LDL-receptor may lead to an increased level of coagulation factor VIII, independent from the level of LDL-C [36].

Lp(a) is known to be pro-coagulant and anti-fibrinolytic, and the evidence is growing that Lp(a) may be a direct cause of CVD [37,38,39]. Lp(a) is an LDL-C macromolecule partly covered with apoprotein (a) (apo(a)). The structure of apo(a) is almost identical to the fibrinolytic pro-enzyme plasminogen, which means that apo(a) impairs the generation of plasmin, which is required for lysis of thrombi. In addition to this ‘anti-fibrinolytic’ property, Lp(a) also stimulates platelet aggregation and promotes endothelial dysfunction [40].

In most papers, measurement of LDL-C consists of aggregating LDL-C and Lp(a). As the cholesterol content of Lp(a) constitutes 30–45% of total LDL-C, it is therefore necessary to correct LDL-C to determine the proportion of Lp(a) [41]. After correction of LDL-C, a study of more than half a million patients found that LDL-C was no longer a risk factor for incident cardiovascular disease [42]. Another study of more than 14,000 individuals noted that Lp(a) was significantly associated with CAC, independent of lipid lowering therapy, conventional cardiovascular risk factors, and pre-existing CVD [43].

Lp(a) may well play the same role in FH. In a study of 388 FH individuals, the CVD hazard ratio for Lp(a) was 2.59 whereas the ratio for LDL-C was 0.85 [44]. In a comparison of 54 FH patients with CVD and 61 without, Lp(a) was significantly higher among those with CVD, whereas LDL-C did not differ [24]. The same was found in a study of 782 FH patients with CVD and 1618 without [45], and in a study of 247 FH patients with CVD and 1713 without [46]. In the latter, LDL-C was even significantly higher among those without CVD.

**Lack of important proteins:** In a study of sixty genetically confirmed FH individuals treated with statin, the participants were stratified into asymptomatisk FH with low atherosclerotic burden (group 1), asymptomatisk FH with high atherosclerotic burden (group 2), and atherosclerotic FH with CVD (group 3) [47]. Using proteomics, the authors identified six new proteins (leucine-rich alpha-2-glycoprotein, inter-alpha-trypsin inhibitor heavy chain H3, complement C4-B, complement C1q subcomponent subunit B, monocyte differentiation antigen, and histidine-rich glycoprotein). Whereas LDL-C did not differ significantly between the three groups, the number of these proteins was about 20 times higher in group 1 than in group 3, and about 10 times higher in group 2 than in group 3.

Lack of these proteins may increase the risk of CVD by various ways. For instance, it has been shown that histidine-rich glycoprotein binds several components of the coagulation and fibrinolysis cascades in mice [48]. As inflammation is considered as a causal factor in atherosclerosis, the authors were surprised because two of the new proteins, which participate in the complement system (complement C4-B and complement C1q subcomponent subunit B), were inversely associated with CVD. However, as mentioned above, there is much evidence that LDL plays an important role in the immune system, and many studies have also shown a strong association between infections and CVD [49]. That FH individuals with CVD had fewer complement factors than those without is also in accordance with the idea that LDL protects against infections.

Furthermore, four meta-analyses of controlled clinical trials including about a million participants have demonstrated that anti-inflammatory treatment using rofecoxib, celecoxib, ibuprofen, diclofenac or other NSAIDs increases the risk of CVD [50,51,52,53]. An apparent counterargument is a recent systematic review and meta-analysis of ten similar trials including about 60,000 patients where the authors found that anti-inflammatory therapy can reduce the incidence of the primary outcome in patients with CAD [54]. However, mortality was unchanged and the risk of infections was increased; thus, the authors ignored the four large meta-analyses with the opposite result mentioned above.

**Diabetes is less frequent among people with FH:** There are more reasons why people with FH live just as long or longer than other people. In a study of 12,300 FH individuals and 24,898 unaffected relatives adjusted for age, body mass index, high-density lipoprotein cholesterol, triglycerides, statin use, smoking, and CVD, the authors found that the number with type 2 diabetes was significantly lower among those with FH [55]. The explanation of this phenomenon is possibly that people with FH had chosen a healthier lifestyle when they became aware of their abnormality. In a cross-sectional study of 2185 adult people with FH and 11,856 individuals from the general population matched for age and sex for instance, those with FH had lower BMI, and fewer of them were or had been smokers than the reference population [56].

**Prevention and treatment**: According to the official guidelines, cholesterol should be lowered as much as possible in FH. Many authorities even suggest that children with FH should lower their cholesterol. However, there is obviously little evidence that high LDL-C is the major cause of premature CVD in FH and no randomized, controlled cholesterol-lowering trial of FH individuals has proved that such treatment is beneficial either. It is therefore highly questionable to use cholesterol-lowering as treatment of people or patients with FH, also because it is well-known that statin treatment has many serious side effects [57]. The commonest one is considered to be muscular damage, but it is little known that many types of cerebrospinal dysfunction may occur as well, both in mice [58] and human beings [57,59,60,61]. They include cognitive dysfunction, suicidal or violent behavior, memory loss, Alzheimer’s, aphasia, polyneuropathy, and many others. However, according to the FDA Adverse Event Reporting System, adverse effects from cerebrospinal dysfunctions are classified in 23 separate reaction terms. Probably, most of them are unusual, but if all of them were to be combined, the total number may be substantial.

The dietary recommendations for people with FH are based on the diet-heart hypothesis. However, there appears to be an almost complete lack of evidence in support of this hypothesis [62,63,64], and no diet is able to change the level of Lp(a) [65]. In accordance with this, several trials have tested various types of diets on FH individuals with no benefits [66]. On the other hand, in a case-report, a physician reported his personal experience with a very-low-carb ketogenic diet, which was able to lower his Lp(a) by about 40% [67]. This effect has recently been supported by a small dietary trial [68]. We think that this is a highly relevant issue for future research.

Many studies have shown that FH individuals who suffer from CVD exhibit the same widely accepted risk factors as other people [64], but we think that it is their thrombogenic factors which alter their additional risk burden. In support of this conjecture are studies on the Watanabe rabbit, which has significantly higher levels of factor VIII and fibrinogen than normal rabbits. Experiments on these FH rabbits have demonstrated that treatment with probucol lowered factor VIII and fibrinogen and prevented atherosclerosis in the absence of any reduction of cholesterol [69]. In another Watanabe experiment, simvastatin treatment lowered cholesterol, but it did not inhibit atherosclerosis [70]. Many clinical trials have shown that probucol is able to improve many types of CVD. Unfortunately, probucol has too many serious adverse effects to be used in clinical practice.

Both observational studies and clinical experiments have shown that proprotein convertase subtilisin/kexin type 9 (PCSK9) inhibitors may produce numerous nonlipid-related pleiotropic effects, such as lowering of fibrinogen, platelet reactivity, and thrombogenesis [71,72,73]. It has therefore been suggested that treatment with PCSK9 inhibitors would be able to lower the risk of CVD among people with FH, but hitherto no experiments with clinical outcome have been published. The chance that such treatment may be beneficial is also questionable, because the PCSK9 inhibitors lower LDL-C even more than the statins, and there is much evidence supporting the idea that high LDL-C is beneficial [2,3,4,5,10,20].

Recently, a randomized, double-blind, one-year long placebo-controlled trial involving 286 patients with established CVD found that a hepatocyte-directed antisense oligonucleotide was able to lower Lp(a) significantly without any serious side effects [74]. Such treatment may possibly be useful in FH individuals with elevated Lp(a) and is a relevant issue for future research.

## 3. Conclusions

A wide range of different studies have shown that high LDL-C is not the main cause of premature CVD among people with FH nor in the general population. There is much evidence that it is only a few people with FH who die prematurely and that the cause in most cases is due to various coagulopathies which they have inherited as well.

## Figures and Tables

**Table 1:** Mean LDL-C and mean age in FH individuals with and without CVD in studies published before the introduction of statin treatment [23,24,25,26] or including non-treated individuals only [27] None of the participants had been treated with other cholesterol lowering drugs either.

Authors	Number		LDL-C (mmol/L)		*p*	Age (Years)		*p*
	CVD+	CVD−	CVD+	CVD−		CVD+	CVD−	
Yamashita et al., 1987 [23] Men Women	23 11	19 13	7.65 8.10	7.09 7.53	ns ns	51.1 56.8	45.9 52.5	ns ns
Seed et al., 1990 [24]	54	61	8.1	8.2	ns	47.6	42.0	<0.05
Hill et al., 1991 [25] Men Women	47 26	68 147	7.13 7.25	6.51 7.01	<0.05 ns	48.2 54.7	45.3 49.7	ns ns
Tato et al., 1993 [26]	32	59	8.14	7.68	ns	50.8	54.8	ns
Jansen et al., 2004 [27]	782	1618	7.45 ^#^	7.37 ^#^	ns	50.4	42.8	<0.001

ns: not significant; ^#^ Measured after at least six weeks without statin treatment.

## Data Availability

Not applicable.
